# Applicability of the Movement Assessment Battery for Children-Second Edition (MABC-2) for Japanese Children Aged 3–6 Years: A Preliminary Investigation Emphasizing Internal Consistency and Factorial Validity

**DOI:** 10.3389/fpsyg.2018.01452

**Published:** 2018-08-31

**Authors:** Shogo Hirata, Yosuke Kita, Masanori Yasunaga, Kota Suzuki, Yasuko Okumura, Hideyuki Okuzumi, Tomio Hosobuchi, Mitsuru Kokubun, Masumi Inagaki, Akio Nakai

**Affiliations:** ^1^Department of Elementary Education, Ibaraki Christian University, Ibaraki, Japan; ^2^Department of Developmental Disorders, National Institute of Mental Health, National Center of Neurology and Psychiatry, Tokyo, Japan; ^3^Department of Occupational Therapy, Kyushu Nutrition Welfare University, Fukuoka, Japan; ^4^Faculty of Education, Tokyo Gakugei University, Tokyo, Japan; ^5^Faculty of Education, Saitama University, Saitama, Japan; ^6^Hyogo Children’s Sleep and Development Medical Research Center, Hyogo, Japan

**Keywords:** Movement Assessment Battery for Children – Second Edition (MABC-2), developmental coordination disorder, motor development, Japanese children, factorial validity

## Abstract

This study investigated the applicability of the Movement Assessment Battery for Children – Second Edition (MABC-2) for 3- to 6-year-old Japanese children, particularly addressing its internal consistency and factorial validity. The MABC-2 test set for 3- to 6-year-old children was administered to 252 children. Differences between Japanese children and those of the original normative sample (i.e. United Kingdom children) were investigated along with sex differences. The Japanese children aged 3–6 years were found to have higher Manual Dexterity and Balance component scores than children of the normative sample. Girls scored higher than boys on the Balance component. Results of several analyses showed good internal consistency of the MABC-2. Confirmatory factor analysis revealed that a theoretical three-component model of the MABC-2 was not fitted to Japanese children aged 3–6 years. Instead, a new three-component model was postulated and discussed. The new three-component model of the MABC-2, with Manual Dexterity, Static Balance and Ball Skills, and Dynamic Balance, has high factorial validity in Japanese children aged 3–6 years.

## Introduction

Developmental coordination disorder (DCD) is a neurodevelopmental disorder characterized by motor performance that is markedly below expected levels, given a person’s chronologic age and earlier opportunities for skill acquisition, none of which is the result of a general medical condition such as cerebral palsy or muscular dystrophy (American Psychiatric Association, 2013). According to DSM-5 (American Psychiatric Association, 2013), poor motor performance might manifest as coordination problems, poor balance, marked delay in achieving developmental motor milestones or in the acquisition of basic motor skills. Actually, DCD is diagnosed with good reliability and validity based on precise standard assessments of motor skills. The Movement Assessment Battery for Children (MABC; Henderson and Sugden, 1992) and its second edition, MABC-2 (Henderson et al., 2007), are standard tests used worldwide for this purpose (Blank et al., 2012). Children with DCD generally show a lower total standard score with this test, indicating that their general level of motor skill is significantly lower than that of typically developing children. Empirical studies conducted using the MABC-2 have also revealed motor skill impairment in children with autism spectrum disorders (ASD) (Whyatt and Craig, 2012; Hirata et al., 2015).

Although MABC-2 has been developed and used widely in English-speaking countries, several researchers have investigated the applicability of MABC-2 for other nations such as Brazil (Valentini et al., 2014) and China (Hua et al., 2013). Nevertheless, few research groups have attempted to create a Japanese version of the MABC-2. To provide reliable diagnosis and treatment of Japanese children with DCD or motor skill impairment, such a version must be created. Therefore, it is necessary to assess the applicability of the MABC-2 for Japanese children to prepare formal standardization of a Japanese version of this test. The MABC-2, which is designed for use with 3- to 16-year-old children, has 24 test items divided into three sets of eight, each intended for use with children of specific ages. The first set of test items, labeled Age Band 1 (AB1), is designed for use with children 3–6 years of age, with Age Band 2 (AB2) for children 7–10 years of age, and Age Band 3 (AB3) for children aged 11–16 years of age. Within each age band, the three-component structure of the MABC-2 is theoretically identical, with Manual Dexterity (MD), Aiming and Catching (AC), and Balance (BAL). The MD component includes three tasks: a one-hand posting task, a timed bimanual assembly task, and an untimed drawing task. The AC component includes tasks requiring the throwing of an object to a target and the catching of an object using both hands. The BAL component includes a static balance task and two dynamic balance tasks such as beam walking and a jumping task. These test items have not been regarded as artificial for non-English-speaking countries such as Brazil (Valentini et al., 2014), Greek (Ellinoudis et al., 2011), China (Hua et al., 2013), and Japan (Kita et al., 2016) because the MABC-2 test items comprised familiar motor tasks that are used frequently during physical activities at nursery schools, elementary schools, and junior high schools. Kita et al. (2016) recently investigated MABC-2 applicability for AB2 in Japanese children, reporting that the MABC-2 for AB2 is suitable for assessing Japanese children based on acceptable internal consistency and high factorial validity. To develop a standardized Japanese version of the MABC-2, psychometric characteristics of the MABC-2 in Japanese children such as internal consistency and factorial validity must be investigated with respect to other age bands such as AB1 and AB3. No report of the relevant literature describes a study investigating the psychometric characteristics of the MABC-2 for AB1 and AB3 in Japanese children. This study investigated the applicability of the MABC-2 for AB1 in Japanese children because investigation of this age band might be helpful for early identification of children with DCD and for intervention on their behalf (Hua et al., 2013).

This study specifically examines two psychometric characteristics of the MABC-2 for AB1: internal consistency and factorial validity. In spite of the worldwide currency of the MABC-2, its reliability and validity of psychological measurement have been criticized (Brown and Lalor, 2009). Several studies have been conducted to ascertain the reliability and validity of the MABC-2, but few have examined its reliability and validity for AB1. The reliability of psychological measurement can be evaluated in terms of criteria such as intra-rater (test–retest) reliability, inter-rater reliability, and internal consistency. To date, two reports have described examination of the inter-rater and intra-rater reliabilities of the MABC-2 for AB1 in Chinese (Hua et al., 2013) and Greek children (Ellinoudis et al., 2011). These studies calculated the intraclass correlation coefficient (ICC) for these purposes, finding high and statistically significant ICCs implying acceptable and excellent inter-rater reliability (ICC of 0.61–0.99; Ellinoudis et al., 2011; Hua et al., 2013) and intra-rater reliability (ICC of 0.83–0.99; Hua et al., 2013). Those are unsurprising results because the MABC-2 consists of concrete and rigid record entry forms. The internal consistency of the MABC-2 for AB1 was also acceptable, but not excellent, based on the value of Cronbach’s alpha coefficients (Ellinoudis et al., 2011; Hua et al., 2013). Kita et al. (2016) reported the necessity of more research to confirm the internal consistency of the MABC-2.

Similarly to reliability, the validity of the MABC-2 was evaluated for several aspects such as content validity, criteria-related validity, and factorial validity. Earlier studies revealed that content-related and criteria-related validity of the MABC-2 for AB1 are excellent and that they are acceptable for Brazilian (Valentini et al., 2014) and Chinese children (Hua et al., 2013). The results of earlier studies, however, are inconsistent with respect to the factorial validity of the MABC-2 for AB1. Schulz et al. (2011) assessed the original standardization sample of the MABC-2 and tested the factorial validity of the MABC-2. As described above, the MABC-2 was assumed to be a three-component model (i.e., MD, AC, and BAL) in each age band. However, Schulz et al. (2011) reported an independent general factor and postulated three motor components: MD, AC, and BAL in 431 children aged 3–6 years (AB1). In their study, this general factor was apparently a motivational factor or a factor reflecting biological maturity related to their growth and physical strength. Hua et al. (2013) also tested the factorial validity of MABC-2 for AB1 in 3- to 6-year-old Chinese children. Those results revealed that a theoretical three-component model with eight test items was also rejected. Results of their confirmatory factor analysis (CFA) revealed that a trimmed three-factor model such as MD, AC, and BAL with six test items (items of “Drawing Trail 1” for MD and “Walking heel raised” for BAL were deleted) is suitable for young Chinese children. In contrast to results reported for these two studies, Ellinoudis et al. (2011) reported that a theoretical three-component model of the MABC-2 is suitable for 3- to 5-year-old Greek children. However, the range of the participants’ ages in the study described by Ellinoudis et al. (2011) was narrower than in either of the other two studies. Based on these mixed findings, the factor structure in a Japanese sample of children aged 3–6 years must be examined to reach a definitive conclusion about the factor structure of the MABC-2 for AB1.

As described above, investigating the internal consistency and factorial validity of the MABC-2 for AB1 in Japanese young children is reasonable. This study, intended as a preliminary investigation preceding formal standardization in Japan, was conducted to investigate the applicability of the MABC-2 for AB1 in Japanese children, particularly addressing its internal consistency and factorial validity. In this study, differences between Japanese children and those of the original normative sample (i.e., United Kingdom children) were also investigated along with sex differences. Kita et al. (2016) reported that Japanese children obtained higher MD and BAL scores than normative children on the original version of the MABC-2 (i.e. United Kingdom children). Moreover, Kita et al. (2016) and Miyahara et al. (1998) reported sex differences in Japanese children’s motor performance detected using the MABC-2 or MABC.

## Materials and Methods

Because of the pilot nature of this study, no strict method of standardization of the psychological test was used, such as back-translation of the test manual or comprehensive sampling that guarantees the representativeness of the sample in terms of the geographic diversity of Japan. Back-translation of the test manual was not conducted in earlier studies (Ellinoudis et al., 2011; Hua et al., 2013; Kita et al., 2016). Furthermore, the parental socioeconomic status, such as educational and income level, was not investigated in this or any earlier study (Ellinoudis et al., 2011; Hua et al., 2013; Kita et al., 2016). Participants’ ethnic origin was also not investigated in this or any earlier Japanese MABC study (Miyahara et al., 1998) because the Japanese population shows higher ethnic homogeneity than those of European or North American countries.

### Participants

This study examined 252 children (112 boys, 140 girls), aged 39–83 months (mean = 62.7 and standard deviation (SD) = 12.3) recruited from four nursery schools in Japan. The schools were located in an urban area (population density > 14,000/km^2^), a suburban area (population density of 3000–4000/km^2^), and two rural areas (population density < 1000/km^2^). The original MABC-2 for AB1 was based on data obtained from 431 children, approximating the proportions of children according to geographical regions of the United Kingdom (Henderson et al., 2007). In general, Japan is classified into eight regions: Hokkaido, Tohoku, Kanto, Chubu, Kinki, Chugoku, Shikoku, and Kyusyu. Three of the four nursery schools participating in this study were in urban, suburban, and rural areas of the Kanto region. The fourth school was in a rural area of the Chugoku region. Children without severe neurological disorder or any physical difficulty participated in this study. These participants had normal or corrected-to-normal vision and had no apparent difficulty that might affect motor skills. These conditions were confirmed from interviews of their guardians. After the test purpose was explained, consent for each child’s participation in the study was obtained from the guardians. All guardians provided written informed consent. Participants were under no obligation to take part in the tests. Only participants who consented to participate freely and voluntarily were included. Ethical approval for the study was obtained from the Research Ethics Board of the Tokyo Gakugei University and the National Center of Neurology and Psychiatry.

### Materials

The Movement Assessment Battery for Children 2 (Henderson et al., 2007) was used. As described above, this test, intended for assessment of children aged 3–16 years, consists of sub-tests that produce a total motor score and three sub-component scores: Manual Dexterity (MD), Aiming and Catching (AC), and Balance (BAL). For this study, the test set for AB1 (3–6 years) was used. The MD component includes three tasks: a one-hand posting task (Posting Coins), a timed bimanual assembly task (Threading Beads), and an untimed drawing task (Drawing Trail 1). The AC component includes tasks requiring the throwing of an object to a target (Throwing Beanbag onto Mat) and the catching of an object using both hands (Catching Beanbag). The BAL component includes a static balance task (One-Leg Balance) and two dynamic balance tasks that involve sustained, controlled movement (Walking Heels Raised), and more explosive action (Jumping on Mat). The original United Kingdom norms were used to convert raw scores to each age-adjusted standard score (SS) such as eight test items, three components, and the total score. The mean of these SSs was 10. The standard deviation was 3. Lower SS values reflect poor motor skill performance.

### Procedures

We received formal permission from the copyright holders of the MABC-2 to create the Japanese version of the MABC-2. After MABC-2 kits were purchased from Pearson Education Limited, the test manual was translated from English into Japanese by two authors of this study (SH and YK). Each had obtained a Ph.D. degree in the field of developmental psychology. Each was familiar with psychological testing for young children. Then, after two independently translated manuals were exchanged (SH read YK’s translated manuals and vice versa), they were confirmed as not being different from one another with respect to the methods of task administration, recording, and scoring of the child’s motor performance. For this study, eight expert psychologists or therapists were fully trained to administer the MABC-2. Each had obtained a Master or Doctorate degree in the field of developmental psychology. They participated as examiners in this study. Each examiner was trained to use the MABC-2 by administering the test for university students and children (not study participants). During this training process, examiners judged that each MABC-2 test item was not artificial for the Japanese children because the MABC-2 for AB1 comprised familiar motor tasks that are used frequently during play at a Japanese nursery school. At each nursery school, the MABC-2 was administered individually to each child in a private room. On average, 25 min were necessary to complete the MABC-2.

### Data analysis

Descriptive statistics such as the mean (M) and SD for each SS of the test item, three-component scores, and the total score were calculated for each age group. To clarify cultural differences (i.e., differences between Japanese children and United Kingdom children aged 3–6 years) in the MABC-2, the mean SSs for the total and three-component scores in this study were subjected to a one-sample *t*-test. Gender differences of the Japanese children in the MABC-2 were also examined using the *t*-test for two independent groups. For these *t*-tests, significance was inferred for *p* < 0.05. The internal consistency was assessed using Cronbach’s alpha coefficients with SSs of the eight test items. Item–Total correlations were also calculated using the SSs of the eight test items and total scores for this purpose. According to earlier studies (Ellinoudis et al., 2011; Hua et al., 2013; Kita et al., 2016), the statistical criteria for Cronbach’s alpha coefficients were set as follows: values of 0.70–0.80 were considered high; values of 0.60–0.69 were considered acceptable; and values less than 0.50 were regarded as having poor reliability. These statistical analyses were conducted using software (IBM SPSS Statistics 22; SPSS Japan Inc., Tokyo).

Confirmatory factor analysis (CFA) was applied using software (IBM SPSS AMOS 22; SPSS Japan Inc., Tokyo) to elucidate the factor structure underlying correlations between tasks of the MABC-2 using the SSs of each test item of AB1. According to earlier studies investigating the factorial validity of the MABC-2, the psychometric model that had the strongest theoretical justification was tested first: the three-component model of the MABC-2. In this model, the three-component factors (i.e., MD, AC, and BAL) affected each specific motor task. For example, the MD component was expected to affect only three motor tasks such as Posting Coins, Threading Beads, and Drawing Trail 1. No double loading was allowed. No correlated measurement error existed, but the factors were expected to correlate (Schulz et al., 2011). To evaluate the propriety of the postulated model, maximum-likelihood estimation methods were used. According to earlier verification of AB2 of the MABC-2 in Japanese children (Kita et al., 2016), multiple fit indices were used to evaluate the model fit. The statistical criteria for good fit between the postulated model and present data were set as follows (Hu and Bentler, 1999): *p* > 0.05 for chi-square values (*x^2^*), the chi-square statistic to degree of freedom ratio (*x^2^/df*) < 5, the Goodness-of-Fit Index (GFI) > 0.95, the Adjusted Goodness-of-Fit Index (AGFI) > 0.95, the Comparative Fit Index (CFI) > 0.95, and the root mean square error of the approximation (RMSEA) < 0.05. Wald tests were used to evaluate the significance of each path coefficient in the postulated model. Significance was inferred for results with *p* < 0.05. This study examined few participants. Therefore, a bootstrapping approach was used to validate our postulated model in accordance with the procedures described by Kita et al. (2016). This analysis was conducted according to the Bollen–Stine procedure (Bollen and Stine, 1992). A total of 2000 bootstrapped samples were generated. Then the postulated model was corrected when the *p*-value was not significant in the Bollen–Stine procedure.

## Results

### Descriptive Statistics

**Table 1** presents means and standard deviations of each SS for eight test items, three components, and a total score for each age. The range of the SSs of each test item was 7.6–11.5; more than half of the scores (71.9%, 23/32 scores) were 9–11. These scores were within the average range of the normal distribution. The SSs for total scores and three components were around 10.0 in all age groups.

**Table 1:** Descriptive statistics of scaled scores.

Age	*n* (%)	Eight Test Items									Three Components		
		Mean (SD)									Mean (SD)		
		Manual Dexterity			Aiming and Catching		Balance						
		Posting Coins	Threading Beads	Drawing Trail 1	Catching Beanbag	Throwing Beanbag onto Mat	One-Leg Balance	Walking Heels Raised	Jumping on Mats	Total Mean (SD)	Manual Dexterity	Aiming and Catching	Balance
3	31 (12.3)	10.6 (2.4)	11.2 (2.8)	7.6 (3.8)	9.7 (3.5)	9.5 (3.4)	9.8 (3.0)	8.7 (3.8)	10.7 (3.2)	9.9 (3.2)	10.2 (2.9)	10.0 (3.2)	9.9 (3.6)
4	79 (31.3)	9.8 (2.8)	10.0 (2.8)	9.1 (3.0)	9.8 (2.9)	7.9 (2.1)	10.3 (3.4)	9.4 (3.3)	10.5 (3.2)	9.7 (3.3)	10.1 (3.1)	9.1 (2.4)	10.4 (3.8)
5	51 (20.2)	9.7 (2.9)	10.3 (3.3)	9.4 (2.7)	11.1 (3.3)	9.0 (3.3)	11.2 (2.5)	9.7 (3.1)	10.3 (3.5)	10.6 (2.7)	10.3 (3.0)	10.4 (3.0)	10.9 (3.3)
6	91 (36.1)	10.8 (2.6)	11.5 (3.2)	9.0 (3.1)	9.6 (3.2)	8.6 (3.4)	11.5 (2.9)	9.5 (2.8)	10.1 (2.7)	10.6 (2.7)	11.3 (3.1)	9.3 (3.1)	10.7 (2.9)
Total	252 (100)	10.2 (2.7)	10.8 (3.1)	9.0 (3.1)	10.0 (3.2)	8.6 (3.1)	10.9 (3.1)	9.4 (3.2)	10.3 (3.1)	10.3 (3.0)	10.6 (3.1)	9.6 (2.9)	10.5 (3.4)

### Cultural and Sex Differences

The average SS for the total score in the present Japanese sample was not significantly different from those in the normative sample [*t* (251) = 1.307, *p* = 0.193]. The average SS for each component score was significantly higher than that in the normative sample with respect to Manual Dexterity [*t* (251) = 2.946, *p* = 0.004] and Balance [*t* (251) = 2.554, *p* = 0.011]. The average SS for the Aiming and Catching was significantly lower than that of normative sample [*t* (251) = -2.344, *p* = 0.02].

**FIGURE 1:**
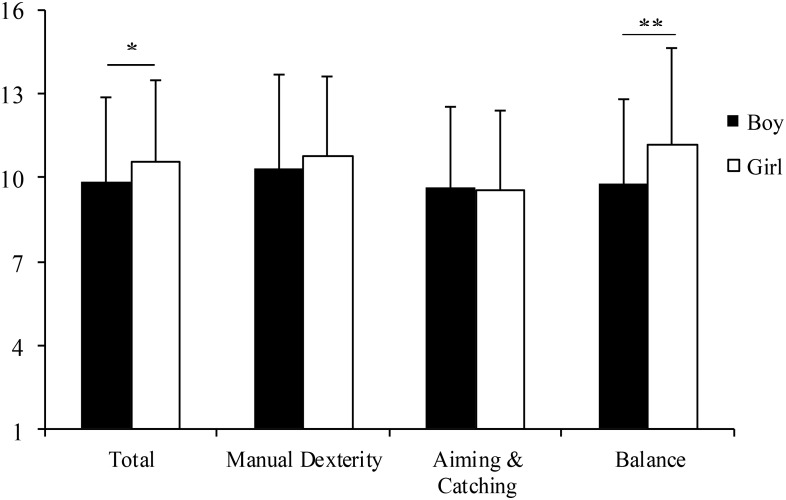
Total and component scores for boys and girls. Bars and error bars, respectively, represent means and SDs. ^∗∗^*p* < 0.01; ^∗^*p* < 0.05.

**Figure 1** presents the average SS for total and each component score for boys and girls in this study. Girls scored significantly higher than boys in the SS for total score [*t* (250) = 1.986, *p* = 0.048] and Balance [*t* (250) = 3.332, *p* = 0.001]. No significant gender difference was found for Manual Dexterity [*t* (250) = 1.112, *p* = 0.267] or Aiming and Catching [*t* (250) = -0.262, *p* = 0.794].

### Internal Consistency

The value of the Cronbach’s alpha coefficient for the eight test items was 0.624, which indicates acceptable internal consistency among the eight test items. **Table 2** presents indexes of internal consistency such as Cronbach’s alpha coefficients after removing each item individually and Item–Total correlations between SSs for eight test items and the total score. Each Cronbach’s alpha coefficient value does not increase gradually (i.e. above 0.1) compared to the overall Cronbach’s alpha coefficient. For Item–Total correlations, almost all values were approximately 0.5–0.6. Correlation was low for Throwing Beanbag onto Mat (*r* = 0.385) and Jumping on Mat (*r* = 0.407). However, all SSs for each test item were correlated significantly with the SS for the total score at *p* < 0.001. These results demonstrate that AB1 of the MABC-2 has acceptable internal consistency.

**Table 2:** Internal consistency of test items.

Items	Cronbach’s alpha coefficient if item deleted	Item-total correlation
Posting Coins	0.576	0.551^∗∗^
Threading Beads	0.568	0.601^∗∗^
Drawing Trail 1	0.588	0.524^∗∗^
Catching Beanbag	0.573	0.578^∗∗^
Throwing Beanbag onto Mat	0.640	0.385^∗∗^
One-Leg Balance	0.564	0.600^∗∗^
Walking Heels Raised	0.600	0.496^∗∗^
Jumping on Mat	0.621	0.407^∗∗^

*Cronbach’s alpha coefficient for the eight test items was 0.624. ^∗∗^*p* < 0.01*.

### Factorial Validity

The theoretical three-component model was rejected clearly according to significant chi-square values [*x^2^*(17) = 39.184, *p* = 0.002; *x^2^/df* = 2.305] and poor fit indices (GFI = 0.961; AGFI = 0.917; CFI = 0.878; RMSEA = 0.072). All path coefficients of test items of 0.232–0.635 were significant, but one test item coefficient (0.232 for Jumping on Mat) was nearly 0.2 (*t* = 2.683, *p* = 0.007). Next, the model described by Schulz et al. (2011), which postulated an independent general factor and three motor components, was tested. However, this model was unidentified. According to these results, the CFA was conducted, moving from a strict confirmatory phase of testing to a more exploratory phase.

First, the test item with the smallest path coefficient, i.e. Jumping on Mat, was dropped. Then the factorial validity of the modified theoretical three-component model of the MABC-2 was tested. Similarly to the first eight-item model, this seven-item model was rejected because its chi-square value was nearly significant [*x^2^*(11) = 19.270, *p* = 0.056; *x^2^/df* = 1.751]. The values of the fit indices were improved, but they remained at an inadequate level (GFI = 0.978; AGFI = 0.944; CFI = 0.949; RMSEA = 0.055). Next, the correlation matrices of the scaled scores (**Table 3**) were checked in accordance with procedures described by Schulz et al. (2011). Correlation between the one-Leg Balance and the Catching Beanbag was strong (*r* = 0.343, *p* = 0.000), but the correlation coefficient for the one-Leg Balance and Jumping on the Mat was nearly 0 (*r* = 0.059, *p* = 0.353). This tendency suggests that the static balance (i.e. One-Leg Balance) and dynamic balance (i.e. Jumping on Mat) differed, and that the static balance is related with ball skills (i.e. Catching Beanbag).

**Table 3:** Correlation matrices of the scaled scores.

Items	1	2	3	4	5	6	7	8
1 Posting Coins	–							
2 Threading Beads	0.411^∗∗^	–						
3 Drawing Trail 1	0.190^∗∗^	0.256^∗^	–					
4 Catching Beanbag	0.243^∗∗^	0.204^∗^	0.246^∗∗^	–				
5 Throwing Beanbag onto Mat	0.136^∗^	0.143	-0.022	0.140^∗^	–			
6 One-Leg Balance	0.264^∗^	0.248^∗∗^	0.195^∗∗^	0.343^∗∗^	0.202^∗∗^	–		
7 Walking Heels Raised	0.108	0.158^∗^	0.219^∗∗^	0.137^∗^	-0.032	0.229^∗∗^	–	
8 Jumping on Mat	0.083	0.102	0.173^∗∗^	0.122	0.003	0.059	0.287^∗∗^	–

*^∗^*p* < 0.05; ^∗∗^*p* < 0.01*.

**FIGURE 2:**
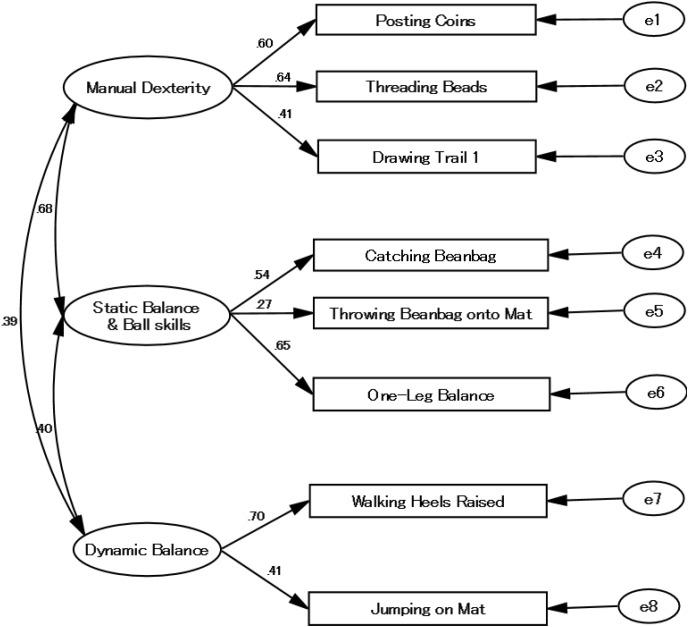
Modified three-component model of MABC-2 for AB1.

Consequently, a new three-component model including criteria such as Manual Dexterity, Static Balance and Ball Skills, and Dynamic Balance (**Figure 2**) was postulated and tested. Results show that the chi-square values of the new three-component model of the MABC-2 were not significant [*x^2^*(17) = 23.455, *p* = 0.135; *x^2^/df* = 1.379] and that the value of fit indices were adequate (GFI = 0.976; AGFI = 0.950; CFI = 0.965; RMSEA = 0.039). All path coefficients of test items of 0.269–0.700 were significant at *p* < 0.05. All components were significantly related to one another. The postulated model was accepted. Therefore, we conducted bootstrapping analysis using 2000 bootstrapped samples. No significant result was found (*p* = 0.169) from bootstrapping analysis using the Bollen–Stine procedure. These results indicate high factorial validity of the new three-component model of the MABC-2 in Japanese children aged 3–6 years.

## Discussion

This pilot study investigated the applicability of the MABC-2 for AB1 in Japanese children aged 3–6 years, particularly addressing its internal consistency and factorial validity. The differences between Japanese children and children of the original normative sample, and sex differences for the MABC-2 for AB1 were also examined. Japanese children aged 3–6 years understood the instructions and performed all test items. It is noteworthy that Japanese children aged 3–6 years obtained higher Manual Dexterity and Balance component scores than the normative children, as did Japanese children aged 7–10 years (Kita et al., 2016). Test items of the MABC differ slightly from those of the MABC-2. Chow et al. (2001) reported similar results to those of this study from MABC scores of Chinese (Hong Kong) children of 4–6 years of age. They reported that Chinese children showed better manual dexterity and balance performance than United States children. Chow et al. (2001) speculated that experience using chopsticks with the dominant hand from early childhood might facilitate manual dexterity performance of other types. Moreover, Chow et al. (2001) report that experience using public transport modes such as buses and boats supports the balance abilities of young children. Most families in Hong Kong use public transport. Therefore, dynamic balance abilities such as jumping on and off of several vehicles are necessary for young children. Young Japanese children also use chopsticks and several modes of public transport such as buses and trains, which require balance ability. Consequently, motor characteristic of young Japanese and Chinese children aged 3–6 years might be results of cultural influences of the eastern Asian region that affect motor development. Cross-cultural investigations must be conducted to clarify this point. From practical perspectives, these results suggest a benefit of generating new norms for Japanese children based on analyses of larger and more representative samples. Kita et al. (2016) pointed out that such efforts are likely to reduce the frequency of misdiagnosis of DCD in Japan.

In this study, girls scored higher than boys with respect to “Balance,” but the genders exhibited similar performance in terms of “Manual Dexterity” and “Aiming and Catching.” Kita et al. (2016) reported that girls scored higher than boys with respect to Balance. Fujiwara et al. (2001) reported significant sex differences in terms of postural movement patterns among Japanese participants aged 18–26 years. Our results suggest that sex differences in balance ability emerged from early childhood in the Japanese population. To verify the sex differences in the balance ability more clearly, sex differences of each balance task were examined using the *t*-test for two independent groups. Girls scored significantly higher than boys in the SS for One-Leg Balance (static balance task) [*t* (250) = 3.281, *p* = 0.001] and Walking Heels Raised (dynamic balance task) [*t* (250) = 2.368, *p* = 0.019]. No significant sex difference was found for Jumping on Mat (dynamic balance task) [*t* (250) = 0.489, *p* = 0.626]. These results suggest that sex differences have no strong influence on the dynamic balance task of the MABC-2. The results might be related to the fact that both Japanese boys and girls use public transport, as suggested earlier. Unfortunately, the sex ratio of participants was not equal in this study. After resolving this point, sex differences in the balance performance must be investigated further.

For this study, internal consistency was assessed using Cronbach’s alpha coefficients and Item–Total correlations. Cronbach’s alpha coefficients for all test items were acceptable in comparison with results of earlier studies (Ellinoudis et al., 2011; Hua et al., 2013; Kita et al., 2016). Item–Total correlation analysis yielded similar results. These results suggest good internal consistency of the MABC-2 for AB1 and adequacy to maintain the existing number of the MABC-2 for AB1 test items.

Results of CFA revealed that a theoretical three-component model of the MABC-2 was not fitted to Japanese children aged 3–6 years. Based on the correlation matrices of the scaled scores, a new three-component model such as one including Manual Dexterity, Static Balance and Ball Skills, and Dynamic Balance was postulated and tested. Multiple fit indices using the CFA indicated high factorial validity of this new three-component model. Bootstrapping analysis also supported the validity of this model, which helped alleviate methodological problems associated with the small number of participants in this study. Therefore, we infer that the new three-component model of the MABC-2 has high factorial validity in Japanese children aged 3–6 years.

Several studies, however, have produced results that do not accord with those of the present study. As described in the section “Introduction,” Ellinoudis et al. (2011) reported a theoretical three-component model of the MABC-2 (MD, AC, and BAL) as suitable for Greek children aged 3–5 years. Kita et al. (2016) also reported that a theoretical three-component model of the MABC-2 was fitted for Japanese children aged 7–10 years. However, some authors have claimed that the factorial validity of the theoretical three-component model of the MABC-2 is debatable (Ellinoudis et al., 2011; Hua et al., 2013). In fact, results of the CFA obtained using an original standardization sample of the MABC-2 have indicated that the simple theoretical three-component model of the MABC-2, i.e., only MD, AC, and BAL components were assumed, was not fitted for children on the AB1 and AB2 (Schulz et al., 2011). Schulz et al. (2011) reported an independent general factor and postulated three motor components for children on the AB1. Therefore, the factor validity of several possible models of MABC-2 in each age band must be compared. Moreover, it is necessary to investigate the developmental changes of the motor component structure for each age. Several authors have claimed that the motor component structure is changed towards differentiation in motor abilities with age (Bruininks and Bruininks, 2005; Schulz et al., 2011). Among our participants, the motor component structure of older children (i.e. 5–6 years old) is likely to fit the theoretical three-component model of the MABC-2 in line with Japanese children aged 7–10 years. It is a point of debate whether the current age bands used in the MABC-2 are appropriate for Japanese children, or not. We expect to address this issue in a future study. For practical purposes, results of the CFA in this study suggest that emphasis should be assigned primarily to the total test score of AB1 for Japanese children, as Schulz et al. (2011) have reported.

In our new three-component model, a distinction became apparent between the task evaluating static and dynamic balance. Tsigilis et al. (2001) reported the possibility that balance is not a general motor ability, but that it is instead assumed to be task-specific. Our results confirm this possibility. Schulz et al. (2011), who assessed the original standardization sample of the MABC-2, reported similar findings from analyses of children aged 7–10 years (AB2). In their results, the static balance factor emerged and became distinguished from the dynamic balance factor. In turn, these two balance factors were integrated to one balance factor in 11- to 16-year-old children (AB3). One might reasonably infer that static balance is related with ball skills such as catching and throwing tasks. Many authors have agreed that balance control is fundamentally important for skilled movement (Chew-Bullock et al., 2012). For instance, Chew-Bullock et al. (2012) reported a significant relation between static balance and kicking accuracy. Moreover, Aalto et al. (1990) demonstrated that experienced rifle shooters had less postural sway during normal standing than untrained participants had. In the Catching Beanbag and Throwing Beanbag tasks of the MABC-2, child instability might engender large differences in performance. To assess this hypothesis, biomechanical analyses must assess the body sway of children when performing ball skills. Japanese therapists must devote attention to the role of static balance in young children’s ball skills.

Finally, this study has several limitations. As explained in the section “Materials and Methods,” the present study did not employ back translation from Japanese to English. Future studies should use back translation to ensure accurate capture of assessment items. Furthermore, several reliability indexes such as intra-rater (test–retest) and inter-rater reliability were not investigated. Our examiners confirmed the content validity of the MABC-2 for Japanese children, but a more objective investigation using a Likert scale is needed. Based on earlier studies described in the section “Introduction” (Ellinoudis et al., 2011; Hua et al., 2013; Kita et al., 2016), we predict that these psychometric indexes of the MABC-2 for AB1 in Japanese children are excellent and acceptable.

## Conclusion

This study investigated the applicability of the MABC-2 for Japanese children aged 3–6 years (AB1). The MABC-2 for AB1 has good internal consistency. Consequently, it is reasonable to maintain the existing number of the MABC-2 for AB1 test items. This result is expected to be beneficial for the next steps for standardization of a Japanese version of the MABC-2. The CFA, however, revealed that a theoretical three-component model of the MABC-2 was not fitted to Japanese children aged 3–6 years. Instead, a new three-component model was proposed. These results suggest that emphasis should be assigned to the total test score of AB1 for Japanese children as a diagnostic tool. For standardization of a Japanese version of the MABC-2, more systematic and comprehensive investigation must be conducted to guarantee the representativeness of the sample in terms of the geographic diversity of Japan. In addition, the intra-rater reliability, inter-rater reliability, and content validity should be confirmed. Moreover, it is necessary to investigate the developmental changes of the MABC-2 factor structure for Japanese children aged 3–16 years.

## Author Contributions

SH, YK, MY, KS, and YO acquired the data. SH and YK analyzed or interpreted the data. SH wrote the manuscript. All the authors made substantial contributions to the conception and design of the study.

## Conflict of Interest Statement

The authors declare that the research was conducted in the absence of any commercial or financial relationships that could be construed as a potential conflict of interest.
